# Effects of cyclopiazonic acid and dexamethasone on serotonin-induced calcium responses in vascular smooth muscle cells

**DOI:** 10.1007/s13105-016-0474-8

**Published:** 2016-03-04

**Authors:** Cigdem Selli, Metiner Tosun

**Affiliations:** Department of Pharmacology, Faculty of Pharmacy, Ege University, 35040 Izmir, Turkey; Applied Bioinformatics of Cancer, Edinburgh Cancer Research Centre, Institute of Genetics and Molecular Medicine, Edinburgh, EH4 2XU UK

**Keywords:** CPA, Dexamethasone, PKC, Serotonin, SOC

## Abstract

We previously observed that sarcoendoplasmic reticulum Ca^2+^ ATPase (SERCA) blockade by cyclopiazonic acid (CPA) significantly potentiates serotonin (5-hydroxytryptamine (5-HT))-induced vascular contractions. Furthermore, 5-HT receptor antagonist methysergide partially inhibited CPA-potentiated 5-HT contractions. In the present study, we further investigated whether SERCA inhibition potentiates 5-HT-induced Ca^2+^ responses along with attenuating the receptor antagonism by store-operated Ca^2+^ (SOC) entry and protein kinase C (PKC)-mediated mechanisms. The effects of dexamethasone that was previously shown to induce SOC entry and enhance 5-HT responses were also tested. For this purpose, intracellular Ca^2+^ levels were monitored in A7r5 embryonic rat vascular smooth muscle cells by spectrofluorometry using the fluorescent indicator fura-2. The results showed that CPA, although not dexamethasone, significantly potentiated 5-HT-induced Ca^2+^ elevations. Ketanserin partially decreased 5-HT-induced and CPA-potentiated Ca^2+^ elevations whereas both PKC inhibitor D-sphingosine and SOC entry blocker 2-aminoethoxydiphenyl borate (2-APB) abolished the remaining responses. The data suggests that diminished antagonistic effect on 5-HT-induced Ca^2+^ elevations in the presence of SERCA inhibition is induced by SOC entry and PKC activation.

## Introduction

Serotonin (5-hydroxytryptamine (5-HT)) is found in the gastrointestinal tract, the central nervous system, and the bloodstream, primarily in platelets. 5-HT released from activated platelets regulates the function of vascular smooth muscle (VSM) cells through activating its G protein-coupled receptors (GPCRs), 5-HT_2A_, and 5-HT_1B_. 5-HT_2A_ receptor activates phospholipase C (PLC) through Gq leading to the accumulation of inositol 1,4,5-trisphosphate (IP_3_) that causes Ca^2+^ release from internal stores and di-acylglycerol (DAG) which activates protein kinase C (PKC) and voltage-operated Ca^2+^ channels [28]. 5-HT-elevated intracellular Ca^2+^ concentration ([Ca^2+^]_i_) has been characterized by two steps, a transient phase due to IP_3_-induced Ca^2+^ release from internal stores and a plateau phase that mainly depends on the extracellular Ca^2+^ influx. Ca^2+^ release from cyclopiazonic acid (CPA)-sensitive intracellular stores and store-operated Ca^2+^ (SOC) entry also contribute to 5-HT-induced responses [25].

SOC entry is activated both by agonist-induced Ca^2+^ store depletion and by sarcoendoplasmic reticulum Ca^2+^ ATPase (SERCA) inhibitors such as thapsigargin and CPA. SERCA is essential to maintain [Ca^2+^]_i_ homeostasis through transporting cytosolic Ca^2+^ into stores besides regulating SOC entry [30]. In addition, SERCA dysfunction has been associated with many cardiovascular disorders including congestive heart failure, cardiac hypertrophy, pathological angiogenesis, as well as atherosclerosis [2]. Balloon injury-induced neointima formation in rat carotid arteries was prevented by SERCA2a gene transfer [23]. The initial report on the deficiency in cardiac SERCA levels in congestive heart failure due to myocardial infarction was published in 1996 [34]. The genetically targeted SERCA2a enzyme replacement therapy, Mydicar®, is currently under clinical investigation for the treatment of advanced heart failure [13].

Switching from SERCA2a to SERCA2b isoform is the key difference between contractile and non-contractile (synthetic/proliferating) VSM phenotypes. It has been known for decades that VSM cells demonstrate phenotypic plasticity with a diverse range of phenotypes even at the differentiated state in contrast to most of other cell types. Although the plasticity is required for vascular development, switching from contractile to synthetic phenotype is also associated with vascular diseases [17]. The role of synthetic VSM cells in atherosclerosis whether they are the key pathogenic factor or beneficial by stabilizing the fibrous cap is still controversial [12]. Restoration of SERCA2a levels was shown to modify agonist-induced Ca^2+^ elevations and SOC entry as well as suppressing human VSM cell proliferation [7]. In accordance with these findings, we previously observed the upregulation of SERCA2b and SOC entry along with suppressed proliferation in A7r5 cells during subculturing [9].

Corticosteroids also result in SERCA inhibition and disruption of Ca^2+^ homeostasis. It is well known that systemic corticosteroid application results in hypertension in normotensive patients and also worsening of blood pressure control in hypertensive patients mainly through increased renal salt and fluid retention. In addition to potentiating vasoconstrictor responses through increased receptor density [24, 26, 35], the actions of corticosteroids on calcium transport of VSM cells including SERCA levels and SOC entry were reported. Administration of triamcinolone, 80 mg/kg for 5 days, reduced SERCA mRNA levels in rat diaphragm suggesting a possible mechanism for fiber atrophy [10]. The synthetic corticosteroid dexamethasone has been shown to activate SOC entry via Ca^2+^-independent phospholipase A_2_ in cultured myotubes, an in vitro model of muscle wasting [18].

SERCA downregulation also alters agonist-induced vascular responses and attenuates receptor antagonism. We previously showed that the inhibitory effects of endothelin-1 (ET-1) and 5-HT receptor antagonists on CPA-potentiated agonist-induced contractions significantly decreased in rat thoracic aorta [32, 33] suggesting the possible SERCA blockade-induced internalization 5HT_2A_ receptors that are localized on caveolar membranes [5]. PKC has been shown to mediate 5-HT-induced 5-HT_2A_ internalization [6] via receptor phosphorylation which is followed by recycling back to the plasma membrane by protein phosphatase 2A (PP2A)-mediated dephosphorylation [27].

Based on these data, we hypothesized that SOC entry and PKC activation are responsible for the decrease in 5-HT receptor antagonism observed in the presence SERCA blockade. To test this hypothesis, we investigated the effects of SOC entry blocker 2-aminoethoxydiphenyl borate (2-APB) and PKC inhibitor (D-sphingosine) on CPA-potentiated and 5-HT-induced Ca^2+^ responses in VSM cells. The abilities of dexamethasone to induce SOC entry and to potentiate 5-HT-induced Ca^2+^ responses in VSM cells were also tested.

## Materials and methods

### Cell culture

A7r5 cells derived from embryonic rat thoracic aorta (European Collection of Cell Cultures (ECACC)) fed with DMEM containing 10 % fetal bovine serum and 2 mM l-glutamine in flasks and maintained in a humidified incubator at 37 °C and 5 % CO_2_. When it reached 70 % confluency, cells were subcultured (1:2) using 0.5 % trypsin-EDTA. A detailed culturing protocol for A7r5 cells has been published recently [9].

### Intracellular Ca^2+^ measurements

Intracellular Ca^2+^ levels were measured on cell populations (at passage numbers 22–24) using a dual wavelength spectrofluorometer (PTI QM8/2005, Photon Technology International, Birmingham, NJ) as described previously [31]. Briefly, A7r5 cells at passage numbers (P#) 22–24 were seeded on round coverslips in 24-well plates at 20,000–30,000 cells/well density and then incubated for 24–48 h to reach a maximum of 70 % confluency. In over confluent cells, spontaneous Ca^2+^ oscillations hindering the monitoring of agonist-induced responses were determined.

For the loading of Ca^2+^ indicator, cells were incubated in HEPES buffered saline (HBS; in millimolar; NaCl 135, KCl 5.9, MgCl_2_ 1.2, CaCl_2_ 1.5, HEPES 11.6, NaHCO_3_ 5.0, glucose 11.5, pH 7.3) containing 5 μM fura-2/AM plus 0.02 % pluronic F-127 (Molecular Probes) supplemented with 1 mg/ml BSA for 1 h at room temperature in the dark. After rinsing twice with HBS containing 1 mg/ml BSA, cells on coverslips were mounted into a polymethacrylate spectrophotometer cuvette with the help of an apparatus. Fluorescence emission at 510 nm was monitored with excitation at 340 and 380 nm and expressed as ratio (340/380). Peak Ca^2+^ responses were evaluated due to time-dependent decays in plateau. Background fluorescence was determined by quenching the fura-2 fluorescence with MnCl_2_ (5 mM) in the presence of 10 μM ionomycin in Ca^2+^-free solution containing 2 mM EGTA at the end of the experiment.

### Chemicals

All chemicals were from Sigma and dissolved in appropriate solvents as follows: 5-HT (PubChem CID: 164531) (10^−2^ M) in distilled water (DW); CPA (PubChem CID: 54695722) (10^−1^ M) in dimethylsulfoxide (DMSO); verapamil (PubChem CID: 155002) (10^−2^ M) in DW, methysergide (PubChem CID:5281073) (10^−2^ M) in DMSO; ketanserin (10^−1^ M) in EtOH; 2-APB (PubChem CID: 1598) (10^−1^ M) in DMSO; D-sphingosine (synthetic) (PubChem CID: 5280335) (10^−2^ M) in EtOH; and dexamethasone (PubChem CID: 5743) (10^−2^ M) in EtOH. In order to avoid direct vasorelaxant effects, final DMSO and EtOH concentrations did not exceed 0.1 %.

### Data analysis

Data analyses as well as graphical presentations were prepared by using GraphPad Prism5. The results were given as mean ± standard error of the mean. “*n*” represents the number of samples used. The significance of differences was evaluated by Student’s *t* test for two groups and one-way ANOVA with post hoc Newman-Keuls test for multiple comparisons. *P* < 0.05 was considered significant.

## Results

### 5-HT-induced Ca^2+^ elevations

We previously showed that non-selective 5-HT receptor antagonist methysergide (1 μM) abolished 5-HT (1 μM)-induced contractions in rat thoracic aorta [32]. In the present study, we further investigated the nature and antagonism of 5-HT-induced Ca^2+^ elevations. The antagonistic effect of methysergide could not be tested due to the disruption of fura-2 fluorescence (data not shown). 5-HT was applied at 1 μM final concentration that previously shown to induce measurable Ca^2+^ elevations in A7r5 cells [9, 31].

**Fig. 1:**
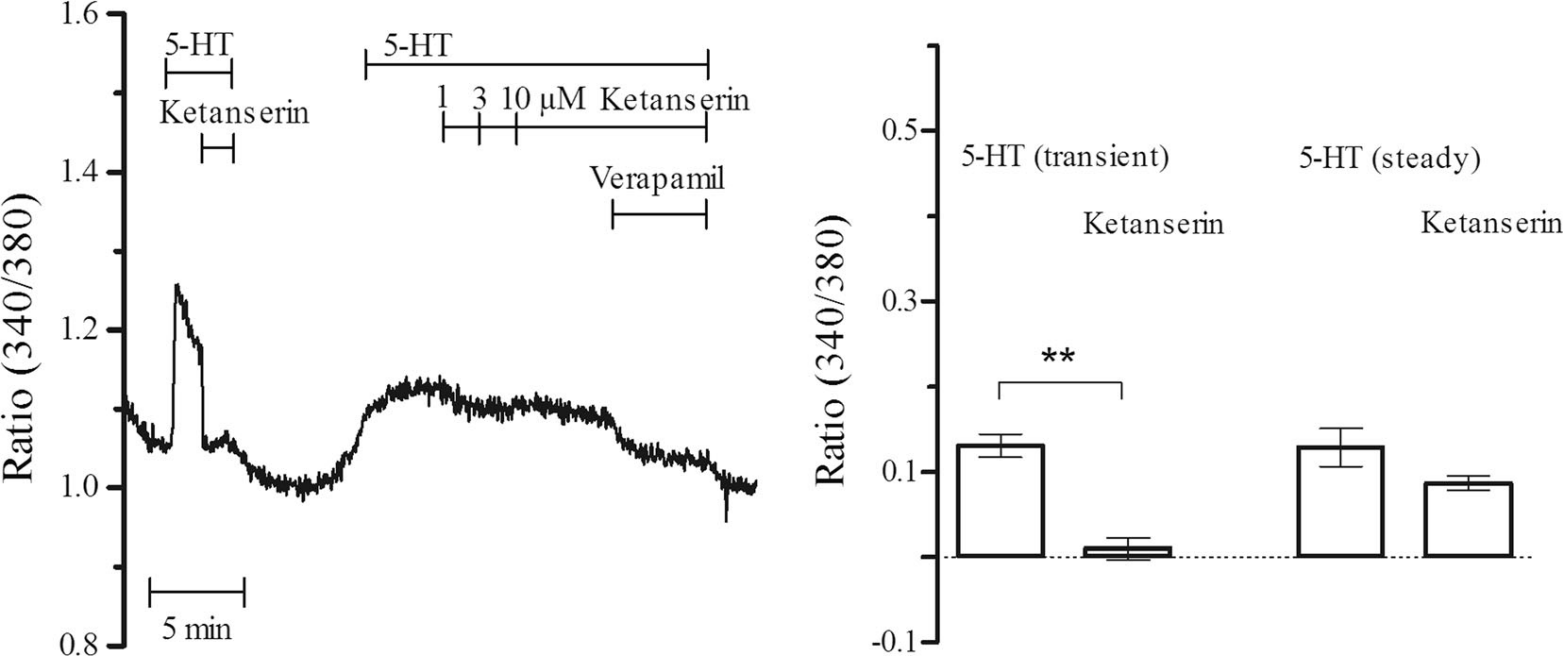
5-HT-induced Ca^2+^ elevations. Transient and steady elevations of Ca^2+^ in response to 5-HT (1 μM) and the effects of ketanserin (1 μM) and verapamil (1 μM) on 5-HT-induced elevations (**P < 0.01, *n* = 3)

Administration of 5-HT resulted in two distinct Ca^2+^ responses: (i) a transient increase that significantly (*P* < 0.01, *n* = 3) and completely (90 %) inhibited by ketanserin (1 μM) and (ii) a steady elevation partially (32 %) reversed by ketanserin (Fig. 1). Figure 1 shows a continuous recording in which the second exposure to 5-HT elicits a steady response that is only weakly inhibited by cumulative doses of ketanserin. The remaining 5-HT steady responses were almost completely inhibited by voltage-operated Ca^2+^ channel blocker verapamil (1 μM).

**Fig. 2:**
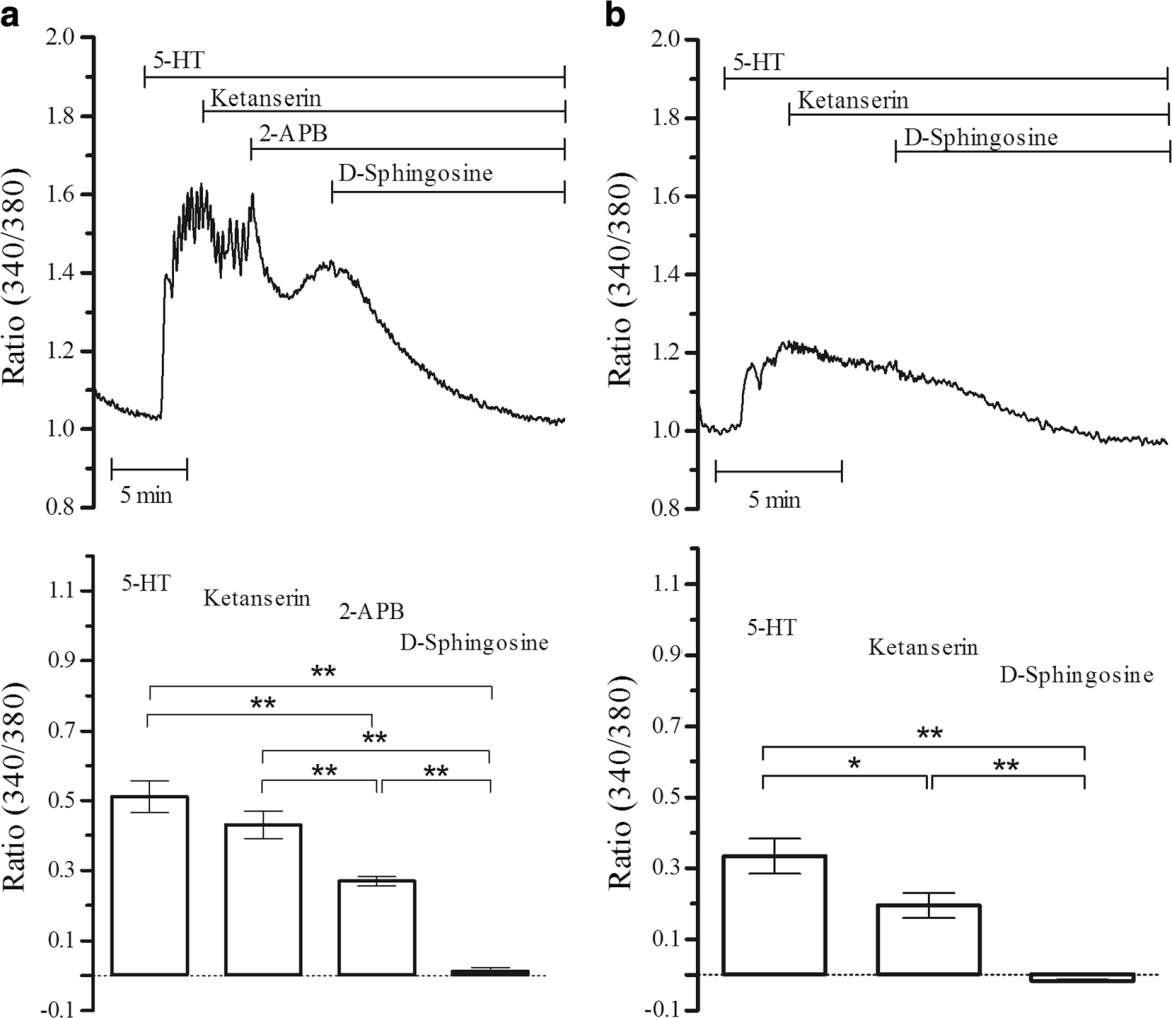
Inhibition of 5-HT-induced Ca^2+^ elevations. **a** 2-APB (50 μM) and D-sphingosine (10 μM) were sequentially applied on ketanserin (1 μM)-inhibited 5-HT (1 μM) responses (**P < 0.01, *n* = 4). **b** D-sphingosine (10 μM) was also administered on ketanserin-inhibited elevations (*P < 0.05, **P < 0.01, *n* = 4)

It is known that 5-HT leads to Ca^2+^ release from CPA-sensitive stores and SOC entry which constitute the first (transient) and second (plateau) phases of 5-HT responses, respectively. In light of this, we further investigated the effects of 2-APB on 5-HT steady elevations. A purported SOC entry blocker 2-APB [31] significantly (*P* < 0.01, *n* = 4) but not completely inhibited the remaining Ca^2+^ elevations (Fig. 2a). Following the observation of the partial inhibition by 2-APB (50 μM), we further investigated the effects of D-sphingosine which is a potent and specific inhibitor of PKC. D-sphingosine (10 μM) abolished (*P* < 0.01, *n* = 4) the remaining responses following 2-APB inhibition (Fig. 2a). The effect of D-sphingosine on ketanserin-inhibited responses was further investigated in the absence of 2-APB (Fig. 2b). Although 5-HT-induced steady Ca^2+^ elevations were significantly (*P* < 0.05) higher in Fig. 2a compared to Fig. 2b, this discrepancy was inevitable in experimental conditions. D-sphingosine (10 μM) abolished (*P* < 0.01, *n* = 4) the responses when applied following ketanserin (Fig. 2b) as well.

### Effects of CPA on 5-HT-induced Ca^2+^ elevations

**Fig. 3:**
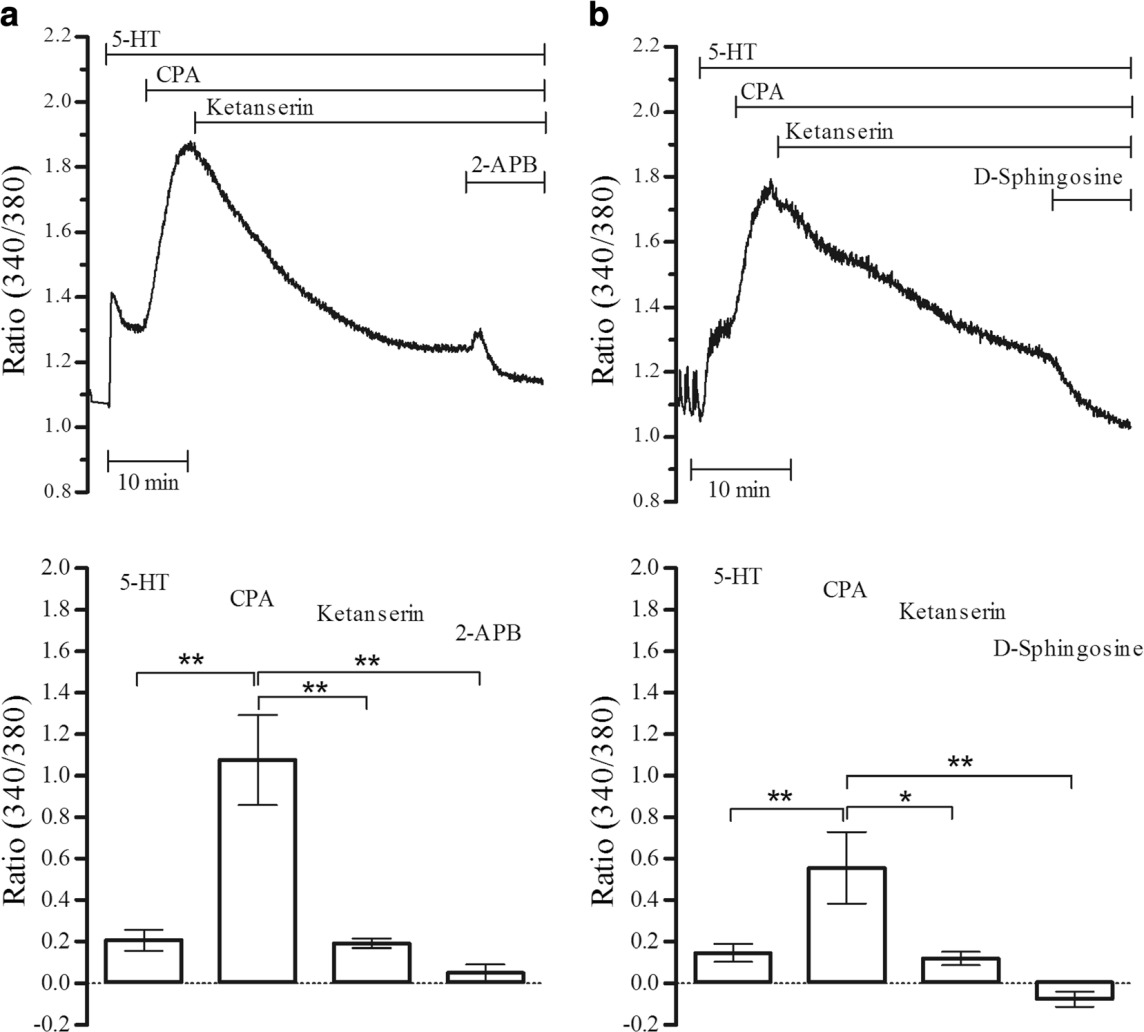
Inhibition of CPA-potentiated 5-HT-induced Ca^2+^ elevations. **a** The effects of ketanserin (1 μM) and 2-APB (50 μM) on CPA (10 μM)-potentiated 5-HT (1 μM) responses (**P < 0.01, *n* = 4). **b** Ketanserin (1 μM) and D-sphingosine (10 μM) were sequentially applied on 5-HT-induced and CPA (10 μM)-potentiated elevations (*P < 0.05, **P < 0.01, *n* = 4)

CPA, at 10 μM concentration that depletes SR-stored Ca^2+^, potentiated 5-HT contractile responses and attenuated 5-HT receptor antagonism in endothelium-denuded rat thoracic aorta [32]. The effects of CPA on 5-HT-induced Ca^2+^ elevations further investigated in the present study. CPA significantly potentiated 5-HT (1 μM)-induced Ca^2+^ responses which were partially inhibited (*P* < 0.05, *n* = 4) by 1 μM ketanserin (Fig. 3). Furthermore, both 2-APB (50 μM) and D-sphingosine (at 10 μM that reportedly inhibits 5-HT receptor internalization [6]) significantly (*P* < 0.01, *n* = 4) reversed the remaining responses (Fig. 3).

### Effects of dexamethasone on 5-HT-induced Ca^2+^ elevations

**Fig. 4:**
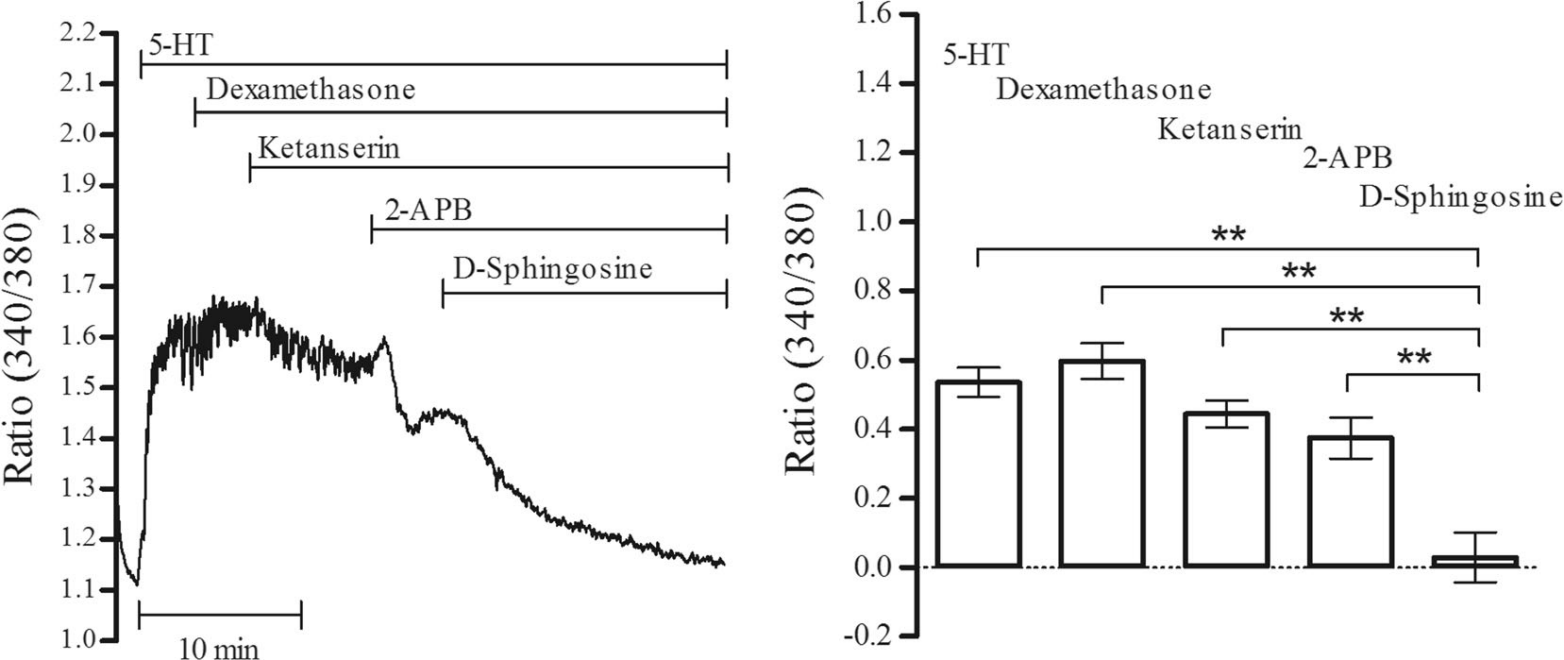
Effects of dexamethasone on 5-HT-induced Ca^2+^ elevations. Dexamethasone (10 μM), ketanserin (1 μM), 2-APB (50 μM), and D-sphingosine (10 μM) were sequentially applied on 5-HT (1 μM)-induced Ca^2+^ responses (**P < 0.01, *n* = 3)

In addition to CPA, the effects of dexamethasone that reportedly activates SOC entry in cultured myotubes [18] were tested. An insignificant increase in 5-HT (1 μM)-induced Ca^2+^ responses was observed with the addition of dexamethasone (10 μM) which was partially inhibited by 1 μM ketanserin and 50 μM 2-APB (Fig. 4). D-sphingosine (10 μM) abolished (*P* < 0.01, *n* = 4) the rest of 5-HT responses (Fig. 4).

## Discussion

We previously showed that 5HT_2A_ receptor antagonist methysergide completely inhibited 5-HT-induced vascular contractions in rat thoracic aorta [32]. However, monitoring the inhibitory effects of methysergide on 5-HT-induced Ca^2+^ elevations in A7r5 cells was not possible due to its spectral properties interfering fura-2 signal. Therefore, another 5-HT_2A_ receptor antagonist ketanserin with potent inhibitory effects on vasoconstrictor action of 5-HT was used. We observed two distinct responses to 5-HT, transient and steady. 5-HT steady responses were served as control for further experiments investigating the effects of CPA and dexamethasone.

### Transient and steady Ca^2+^ responses to 5-HT

During VSM phenotypic alteration, Ca^2+^ handling switches from voltage-sensitive to SOC and receptor-operated Ca^2+^ entries and also Ca^2+^ transients are replaced with steady-state Ca^2+^ elevations [4]. The mode of intracellular Ca^2+^ elevations also determines the type of VSM contraction. Oscillatory type of Ca^2+^ transient triggers phasic contractions whereas a steady-state increase in Ca^2+^ triggers tonic contractions which had been observed in synthetic phenotype [1]. Ketanserin-sensitive and transient Ca^2+^ elevations to 5-HT were previously reported in primary cultured smooth muscle cells [20]. We observed both transient and profoundly steady Ca^2+^ elevations in VSM cell line. Steady 5-HT responses were partially sensitive to ketanserin in contrast to transient elevations which were completely inhibited by ketanserin. 5-HT-induced steady Ca^2+^ elevations observed in our study may be responsible for tonic vasoconstriction supporting its role in pulmonary hypertension [8], and steady elevations may also account for the partial reduction of pulmonary vascular resistance by ketanserin [15]. In cultured rat aortic smooth muscle cells, dose-dependent increases in 5-HT-induced intracellular Ca^2+^ elevations were inhibited by ketanserin [16]. However, in another study performed with the same cell type ketanserin, along with cinanserin and mianserin, it was found to be less effective on 5-HT-induced Ca^2+^ elevations in comparison to sarpogrelate [29]. The discrepancies in 5-HT responses and 5-HT receptor antagonism even in the same cell population may result from spatiotemporal changes in Ca^2+^ signaling and related protein localization. Since restoring SERCA2a levels in synthetic VSM cells modifies the type of 5-HT-induced Ca^2+^ transients from steady-state to transient mode [7], differential localization of SERCA2a may be responsible for two distinct 5-HT responses observed in our study. We also previously reported that culturing conditions and passaging alter the expression of Ca^2+^-related proteins in A7r5 cells that mimic the phenotypic switching of VSM cells [9]. The discrepancies in ketanserin responses observed in different studies may result from different passage numbers and cell culturing conditions. The observation of both ketanserin-sensitive and ketanserin-insensitive 5-HT responses in the present study may also support the utility of A7r5 in the investigation of VSM cell differentiation.

In addition, the steady 5-HT responses were partially (30 %) reversed by ketanserin whereas completely inhibited both by 2-APB and D-sphingosine suggesting the contribution of SOC entry and PKC activation in altered 5-HT-induced Ca^2+^ mobilization. 5-HT-induced steady Ca^2+^ response may result in PKC-mediated phosphorylation and sequential internalization of receptor resulting in decreased antagonistic effect.

### SERCA inhibition potentiates 5-HT-induced Ca^2+^ responses

Enhancement of contractile responses to 5-HT is related to cardiovascular diseases, such as atherosclerosis. Endothelial dysfunction is characterized by reduced endothelium-dependent relaxations and an early marker of atherosclerosis, a chronic inflammatory disease affecting the peripheral arteries and the aorta. Endothelial dysfunction also contributes to increased vasoconstriction induced by endothelium-dependent agonists such as 5-HT. Contractile responses to 5-HT, a major product of platelet activation, were potentiated in coronary arteries of atherosclerotic monkeys and reversed to normal by reducing dietary cholesterol [21]. Furthermore, 5-HT has constricting effects contributing to myocardial ischemia and coronary artery disease in contrast to its vasodilating effect on normal human coronary arteries [11]. It is also known that acute hypertension potentiates 5-HT constrictor responses in coronary arteries by impairing endothelial function [22] along with the enhancement of 5-HT-stimulated [Ca^2+^]_i_-dependent thoracic and abdominal aortic contractions in chronic hypertension [3].

We observed that CPA-mediated SERCA inhibition that mimics impaired SERCA activity which is also a characteristic of synthetic VSM cells [23] potentiated 5-HT-induced Ca^2+^ elevations. When applied alone, CPA (10 μM) causes contraction of endothelium-denuded rat thoracic aorta as well as elevating [Ca^2+^]_i_ in VSM cells [31]. In addition to its PKC-mediated contractile effects, CPA was shown to augment 5-HT- and ET-1-induced vasoconstrictions and to diminish inhibitory actions of their receptor blockers [32, 33]. In accordance with our previous findings in rat aortic rings, SERCA inhibition potentiated 5-HT-induced Ca^2+^ elevations in VSM cells. In contrast to enhanced 5-HT responses observed in our study, SERCA blockers thapsigargin and cyclopiazonic acid were previously shown to inhibit 5-HT-induced Ca^2+^ elevations in rat aortic smooth muscle cells [29].

Since we previously reported that verapamil had no effect on CPA-induced Ca^2+^ elevations [31], the CPA response appears to be related to SOC entry without the contribution of voltage-operated Ca^2+^ channels. Ketanserin partially decreased CPA-potentiated 5-HT-induced Ca^2+^ elevations whereas both PKC inhibitor D-sphingosine and SOC blocker 2-APB abolished the remaining of ketanserin-inhibited responses. This suggests that SERCA blockade-induced SOC entry contributes to the increased Ca^2+^ levels and activation of PKC. It was reported that PKC-mediated pathway rather than the release of stored Ca^2+^ plays a primary role in 5-HT- and histamine-induced coronary spasm [19]. PKC was also suggested to have a role in the recycling of 5-HT_2A_ receptors [6]. These results support that the decrease in 5-HT receptor antagonism in the presence of CPA may result from PKC-mediated internalization of 5-HT_2A_ receptors which have been shown to localize on caveolar membranes [5]. Since sphingosine reportedly counteracts PKC’s effects by regulating PP2A [14], it may facilitate the translocation of internalized 5-HT_2A_ receptors back to the cellular membrane making them available for antagonistic intervention. However, further investigation is required to confirm 5-HT receptor localization using imaging techniques such as total internal reflection fluorescence (TIRF) that are well suited for visualizing single molecules.

### Dexamethasone and 5-HT-induced Ca^2+^ elevations

Enhanced vascular sensitivity to 5-HT through increased mobilization of Ca^2+^ from cellular stores was observed in femoral arteries of deoxycorticosterone acetate (DOCA)-salt hypertensive rats [24]. In another study performed with C6 glioma cells, dexamethasone was shown to potentiate 5-HT-induced responses with a ninefold leftward shift in 5-HT dose-response curves [26]. Furthermore, ketanserin completely inhibited 5-HT-induced time- and dose-dependent increases in [Ca^2+^]_i_ as well as those potentiated by dexamethasone [26]. Dexamethasone was shown to induce the rapid promotion of norepinephrine-mediated rat VSM cell contraction as well [35]. In addition, dexamethasone activates SOC entry and protein degradation in myotubes suggesting the possible involvement of SOC entry in glucocorticoid-induced muscle protein degradation [18].

In contrast to these findings, dexamethasone induced a slight increase in 5-HT-induced Ca^2+^ elevations in our study. In the presence of dexamethasone, the attenuation of the antagonistic effect of ketanserin on 5-HT-induced Ca^2+^ elevations was similar to control responses (26 and 16 %, respectively). However, 5-HT responses in the presence of dexamethasone were not significantly inhibited by 2-APB in contrast to the control (37 and 47 %, respectively) suggesting the contribution of SOC entry-independent mechanisms to dexamethasone-induced Ca^2+^ elevations in A7r5 cells. The results may help to clarify the differential effects of CPA and dexamethasone, both agents activate SOC entry with different mechanisms and efficiencies, on 5-HT-evoked responses.

In conclusion, our findings suggest that decreased 5-HT receptor antagonism in the presence of SERCA inhibition is mediated by SOC entry and PKC activation. Furthermore, 5-HT-induced steady Ca^2+^ elevations may facilitate tonic vasoconstriction associated with vasospastic diseases. Our results may contribute to the elucidation of the underlying mechanisms and the treatment of vascular diseases related to SERCA downregulation and 5-HT hyperreactivity. The attenuated 5-HT receptor antagonism in the presence of SERCA inhibition observed in our study may mimic the phenotypic switching of VSM cells and alter Ca^2+^ signaling in some vascular diseases [17]. However, the major limitation of our study is the lack of physiopathological context. Therefore, the functional role of the attenuation of 5-HT receptor antagonism and SERCA downregulation needs further investigation using cardiovascular disease models.

## References

[1] Aalkjaer C, Nilsson H (2005). Vasomotion: cellular background for the oscillator and for the synchronization of smooth muscle cells. Br J Pharmacol.

[2] Adachi T (2010). Modulation of vascular sarco/endoplasmic reticulum calcium ATPase in cardiovascular pathophysiology. Adv Pharmacol.

[3] Bell DR (1995). Effect of chronic high-pressure on transient and tonic vascular contractions to serotonin in hypertension. Am J Hypertens.

[4] Berra-Romani R, Mazzocco-Spezzia A, Pulina MV, Golovina VA (2008). Ca2+ handling is altered when arterial myocytes progress from a contractile to a proliferative phenotype in culture. Am J Physiol Cell Physiol.

[5] Bhatnagar A, Sheffler DJ, Kroeze WK, Compton-Toth B, Roth BL (2004). Caveolin-1 interacts with 5-HT2A serotonin receptors and profoundly modulates the signaling of selected Galphaq-coupled protein receptors. J Biol Chem.

[6] Bhattacharyya S, Puri S, Miledi R, Panicker MM (2002). Internalization and recycling of 5-HT2A receptors activated by serotonin and protein kinase C-mediated mechanisms. Proc Natl Acad Sci U S A.

[7] Bobe R, Hadri L, Lopez JJ, Sassi Y, Atassi F, Karakikes I, Liang L, Limon I, Lompre AM, Hatem SN, Hajjar RJ, Lipskaia L (2011). SERCA2a controls the mode of agonist-induced intracellular Ca2+ signal, transcription factor NFAT and proliferation in human vascular smooth muscle cells. J Mol Cell Cardiol.

[8] Egermayer P, Town GI, Peacock AJ (1999). Role of serotonin in the pathogenesis of acute and chronic pulmonary hypertension. Thorax.

[9] Erac Y, Selli C, Filik P, Tosun M (2014). Effects of passage number on proliferation and store-operated calcium entry in A7r5 vascular smooth muscle cells. J Pharmacol Toxicol Methods.

[10] Gayan-Ramirez G, Vanzeir L, Wuytack F, Decramer M (2000). Corticosteroids decrease mRNA levels of SERCA pumps, whereas they increase sarcolipin mRNA in the rat diaphragm. J Physiol Lond.

[11] Golino P, Piscione F, Willerson JT, Cappellibigazzi M, Focaccio A, Villari B, Indolfi C, Russolillo E, Condorelli M, Chiariello M (1991). Divergent effects of serotonin on coronary-artery dimensions and blood-flow in patients with coronary atherosclerosis and control patients. N Engl J Med.

[12] Gomez D, Owens GK (2012). Smooth muscle cell phenotypic switching in atherosclerosis. Cardiovasc Res.

[13] Greenberg B, Yaroshinsky A, Zsebo KM, Butler J, Felker GM, Voors AA, Rudy JJ, Wagner K, Hajjar RJ (2014). Design of a phase 2b trial of intracoronary administration of AAV1/SERCA2a in patients with advanced heart failure: the CUPID 2 trial (calcium up-regulation by percutaneous administration of gene therapy in cardiac disease phase 2b). JACC Heart Fail.

[14] Habrukowich C, Han DK, Le A, Rezaul K, Pan W, Ghosh M, Li ZG, Dodge-Kafka K, Jiang XJ, Bittman R, Hla T (2010). Sphingosine interaction with acidic leucine-rich nuclear phosphoprotein-32A (ANP32A) regulates PP2A activity and cyclooxygenase (COX)-2 expression in human endothelial cells. J Biol Chem.

[15] Hamet A, Kral B, Cernohorsky D (1985). Comparative effects of oxygen, nifedipine and ketanserin in hypoxic pulmonary hypertension. Cor Vasa.

[16] Hirafuji M, Nezu A, Kanai Y, Ebihara T, Kawahara F, Tanimura A, Minami M (1998). Effect of 5-hydroxytryptamine on intracellular calcium dynamics in cultured rat vascular smooth muscle. Res Commun Mol Pathol Pharmacol.

[17] Potier M, Bisaillon J, Singer HA, Trebak M, House SJ (2008). The non-excitable smooth muscle: calcium signaling and phenotypic switching during vascular disease. Pflugers Arch.

[18] Itagaki K, Menconi M, Antoniu B, Zhang Q, Gonnella P, Soybel D, Hauser C, Hasselgren PO (2010). Dexamethasone stimulates store-operated calcium entry and protein degradation in cultured L6 myotubes through a phospholipase A(2)-dependent mechanism. Am J Physiol Cell Physiol.

[19] Kadokami T, Shimokawa H, Fukumoto Y, Ito A, Takayanagi T, Egashira K, Takeshita A (1996). Coronary artery spasm does not depend on the intracellular calcium store but is substantially mediated by the protein kinase C-mediated pathway in a swine model with interleukin-1 beta in vivo. Circulation.

[20] Kanaide H, Hasegawa M, Kobayashi S, Nakamura M (1987). Serotonin-induced cytosolic free calcium transients in cultured vascular smooth-muscle cells. Biochem Biophys Res Commun.

[21] Lamping KG, Piegors DJ, Benzuly KH, Armstrong ML, Heistad DD (1994). Enhanced coronary vasoconstrictive response to serotonin subsides after removal of dietary-cholesterol in atherosclerotic monkeys. Arterioscler Thromb.

[22] Lamping KG, Dole WP (1987). Acute hypertension selectively potentiates constrictor responses of large coronary arteries to serotonin by altering endothelial function in vivo. Circ Res.

[23] Lipskaia L, del Monte F, Capiod T, Yacoubi S, Hadri L, Hours M, Hajjar RJ, Lompre AM (2005). Sarco/endoplasmic reticulum Ca2 + -ATPase gene transfer reduces vascular smooth muscle cell proliferation and neointima formation in the rat. Circ Res.

[24] Mecca TE, Webb RC (1984). Vascular-responses to serotonin in steroid hypertensive rats. Hypertension.

[25] Moneer Z, Pino I, Taylor EJ, Broad LM, Liu Y, Tovey SC, Staali L, Taylor CW (2005). Different phospholipase-C-coupled receptors differentially regulate capacitative and non-capacitative Ca2+ entry in A7r5 cells. Biochem J.

[26] Muraoka SI, Mikuni M, Kagaya A, Saitoh K, Takahashi K (1993). Dexamethasone potentiates serotonin-2 receptor-mediated intracellular Ca2+ mobilization in C6 glioma cells. Neuroendocrinology.

[27] Raote I, Bhattacharyya S, Panicker MM (2013). Functional selectivity in serotonin receptor 2A (5-HT2A) endocytosis, recycling, and phosphorylation. Mol Pharmacol.

[28] Rodat-Despoix L, Aires V, Ducret T, Marthan R, Savineau JP, Rousseau E, Guibert C (2009). Signalling pathways involved in the contractile response to 5-HT in the human pulmonary artery. Eur Respir J.

[29] Saini HK, Sharma SK, Zahradka P, Kumamoto H, Takeda N, Dhalla NS (2003). Attenuation of the serotonin-induced increase in intracellular calcium in rat aortic smooth muscle cells by sarpogrelate. Can J Physiol Pharmacol.

[30] Satoh K, Matsu-Ura T, Enomoto M, Nakamura H, Michikawa T, Mikoshiba K (2011). Highly cooperative dependence of sarco/endoplasmic reticulum calcium ATPase (SERCA) 2a pump activity on cytosolic calcium in living cells. J Biol Chem.

[31] Selli C, Erac Y, Kosova B, Tosun M (2009). Post-transcriptional silencing of TRPC1 ion channel gene by RNA interference upregulates TRPC6 expression and store-operated Ca2+ entry in A7r5 vascular smooth muscle cells. Vasc Pharmacol.

[32] Selli C, Erac Y, Tosun M (2014). Cyclopiazonic acid alters serotonin-induced responses in rat thoracic aorta. Vasc Pharmacol.

[33] Tosun M, Erac Y, Selli C, Karakaya N (2006). Sarcoplasmic-endoplasmic reticulum Ca2+-ATPase inhibition prevents endothelin A receptor antagonism in rat aorta. Am J Physiol Heart Circ Physiol.

[34] Zarain-Herzberg A, Afzal N, Elimban V, Dhalla NS (1996). Decreased expression of cardiac sarcoplasmic reticulum Ca(2+)-pump ATPase in congestive heart failure due to myocardial infarction. Mol Cell Biochem.

[35] Zhang T, Shi WL, Tasker JG, Zhou JR, Peng YL, Miao CY, Yang YJ, Jiang CL (2013). Dexamethasone induces rapid promotion of norepinephrine-mediated vascular smooth muscle cell contraction. Mol Med Rep.

